# Automated Diagnosis of Canine Gastrointestinal Parasites Using Image Analysis

**DOI:** 10.3390/pathogens9020139

**Published:** 2020-02-20

**Authors:** Sandra Valéria Inácio, Jancarlo Ferreira Gomes, Alexandre Xavier Falcão, Celso Tetsuo Nagase Suzuki, Walter Bertequini Nagata, Saulo Hudson Nery Loiola, Bianca Martins dos Santos, Felipe Augusto Soares, Stefani Laryssa Rosa, Carolina Beatriz Baptista, Graziella Borges Alves, Katia Denise Saraiva Bresciani

**Affiliations:** 1 School of Veterinary Medicine, São Paulo State University, Araçatuba, São Paulo 16050-680, Brazil; sandra.valeria@unesp.br (S.V.I.); walter.bn@hotmail.com (W.B.N.); carolina.beatriz@unesp.br (C.B.B.); graziella.b.alves@unesp.br (G.B.A.); 2 School of Medical Sciences, University of Campinas, São Paulo 13083-887, Brazil; jgomes@ic.unicamp.br (J.F.G.); s228127@dac.unicamp.br (S.H.N.L.); b120382@dac.unicamp.br (B.M.d.S.); f153466@dac.unicamp.br (F.A.S.); s192512@dac.unicamp.br (S.L.R.); 3 Laboratory of Image Data Science, Institute of Computing, University of Campinas, São Paulo 13083-852, Brazil; afalcao@ic.unicamp.br (A.X.F.); celso@lids.ic.unicamp.br (C.T.N.S.)

**Keywords:** Automation, diagnosis, dogs, parasites, stool

## Abstract

Because canine intestinal parasites are considered cosmopolitan, they carry significant zoonotic potential to public health. These etiological agents are routinely diagnosed using microscopic examination commonly used because of its low cost, simple execution, and direct evidence. However, there are reports in the literature on the poor performance of this test due to low to moderate sensitivity resulting from frequent errors, procedures and interpretation. Therefore, to improve the diagnostic efficiency of microscopic examination in veterinary medicine, we developed and evaluated a unique new protocol. This system was tested in a study involving four genera of highly prevalent canine intestinal parasites in an endemic region in São Paulo state, Brazil. Fecal samples from 104 animals were collected for this research. The new protocol had a significantly higher (*p* < 0.0001) number of positive cases on image data, including parasites and impurities, and was elaborate to test them with the TF-GII/Dog technique, with a moderate agreement and Kappa index of 0.7636. We concluded that the new Prototic Coproparasitological Test for Dogs (PC-Test Dog) allowed a better visualization of the parasitic structures and showed a favorable result for the diagnosis of intestinal parasites in dogs.

## 1. Introduction

Because canine intestinal parasites are considered cosmopolitan, they present a significant zoonotic threat to public health [1,2]. In regions of developing countries with tropical, subtropical, and equatorial climates, four genera of parasites among metazoan and protozoan stand out as highly prevalent in dog infection: *Toxocara* spp., *Ancylostoma* spp., *Trichuris* spp., and *Giardia* spp. [2,3,4].

Among the three genera of multicellular parasites mentioned above, the *Toxocara canis* species is the most prevalent in pet dogs, presenting as the most frequent clinical symptoms diarrhea, flatulence, abdominal distension, dehydration, and developmental delay [5,6]. This parasitic species is considered of great epidemiological importance because it enables the transmission of Visceral Larva Migrans (VLM) and the special variant, Ocular Larva Migrans (OLM), between dogs and humans.

Although less prevalent than *Toxocara* spp. in developing countries, infection with the parasite *Ancylostoma* spp. can cause serious organic problems in dogs and humans [2]. In dogs, symptoms of infection with this parasite are intermittent diarrhea, anemia, and dehydration, followed by death, especially in young animals [7,8]. In turn, humans may be affected by the Cutaneous Larva Migrans (CLM), which causes a pathological condition known as creeping eruption, a dermatitis resulting from the accidental contact of an individual with an infective larval biological phase of the canine parasite *Ancylostoma* spp. 

The multicellular species *Trichuris vulpis* can cause considerable abdominal pain, protein loss, inflammation, intestinal bleeding, and diarrhea in infected dogs. Depending on the intensity of infection and animal immunological capacity, this parasite can cause severe anemia resulting from iron deficiency [1,9,10].

Regarding the genus of protozoan *Giardia* spp., the eukaryote species *Giardia duodenalis* is considered of great anthropozoonotic importance by the World Health Organization (WHO) and is effectively subdivided into different genotypic groups. Infection with this enteroparasite has diarrhea as a common symptom in humans and in more than twenty breeds of vertebrate animals, including dogs [11,12]. The clinical manifestation resulting from this parasitic infection may range from acute to chronic, with intermittent peaks and irregular elimination of cysts in the feces of a vertebrate host [13,14,15].

Parasites of the intestinal tract of dogs are routinely diagnosed by microscopic examination of the fecal samples, mainly because of its the low cost, simple execution, and direct evidence [3,7,16,17,18,19,20]. Immunological, direct and indirect, and molecular methods have been employed in epidemiological surveys and scientific research for characterizing the parasite species, respectively, but with serious restrictions on the routine use of a public and private clinical analysis laboratory, especially when related to high costs and false diagnostic results [14,19,21]. The nonconformity analysis using these methods may result from several circumstances, among them, antigenic variation of the pathogen, cross-reactions, exacerbated host response against parasite epitopes, and contamination. The scientific literature recommend that the use of these methods should be restricted to government programs and academic research [14,22,23,24].

Despite the benefits previously mentioned for routine laboratory microscopy examination, there are reports in the literature that this test has limited effectiveness, resulting from low to moderate diagnostic sensitivity. According to several authors [3,14,21,23], the unsatisfactory diagnostic value of this exam is related to two types of frequent errors: processing and interpretation. The processing error results from errors that may occur during several stages, from field sampling to the preparation of fecal material in the laboratory, while misinterpretation may result from microscopy professionals being poorly trained to broadly identify the parasite species [14,24].

For about fifteen years a parasitological technique known as the Three Fecal Test (TF-Test) has been used with significant results in several academic studies in Brazil [3,14,17,19,21,23,25]. Comparisons among conventional techniques that use fecal samples from small and large animals and humans indicated that the TF-Test has a higher diagnostic sensitivity because it uses appropriate bottles containing preservative solution for the different stages, triple sampling, homogenization, and transport of stools, as well as an adequate laboratory protocol, which decreases the procedural errors considerably [21].

**Figure 1:**
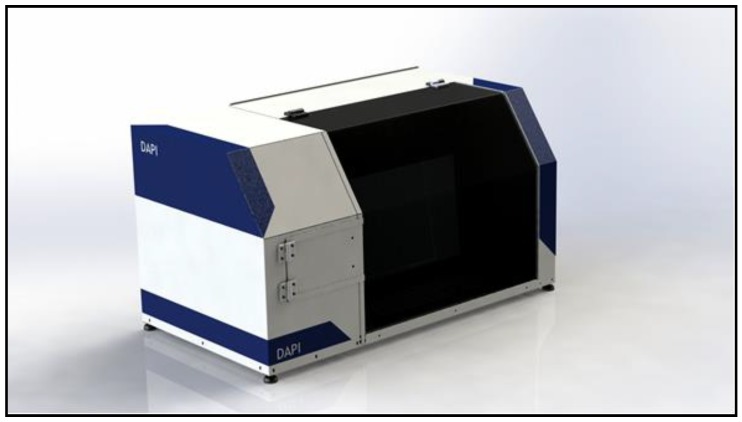
Front/side images of the “Automated Diagnosis of Intestinal Parasites” (DAPI) system, equipped with an internal computer, digital camera, optical tube, and motorized stage.

Currently, a broad scientific and technological study for automated diagnosis is being developed in the Health Medicine and Computer Science areas at the University of Campinas (UNICAMP) in Brazil, seeking to reduce the two types of errors previously described, and consists of a parasitological protocol, computer and customized equipment containing a high-resolution digital camera, appropriate optical tube, and motorized stage [26,27,28,29]. This new system is named “Automated Diagnosis of Intestinal Parasites” (acronym in Portuguese, DAPI) (Figure 1).

We aimed in this effective study to develop and evaluate new protocol to detect four genera of highly prevalent canine intestinal parasites in an endemic region of São Paulo state, Brazil, and to compare it with visual diagnosis and automatic recognition after a coproscopy by the flotation method using zinc sulfate using the TF-GII/Dog and Prototic Coproparasitological (PC)-TEST DOG technique.

## 2. Materials and Methods

### 2.1. Study Description 

The 104 dogs used in this study were of no defined breed, gender, and age (puppies to adults), and they came from shelters located in Araçatuba, SP, an endemic region for canine parasitic diseases in São Paulo State, Brazil. The study was approved by the Animal Experimentation and Ethics Committee (CEEA) of the School of Dentistry (FO) of UNESP, Araçatuba, SP, protocol 00968-2017.

**Figure 2:**
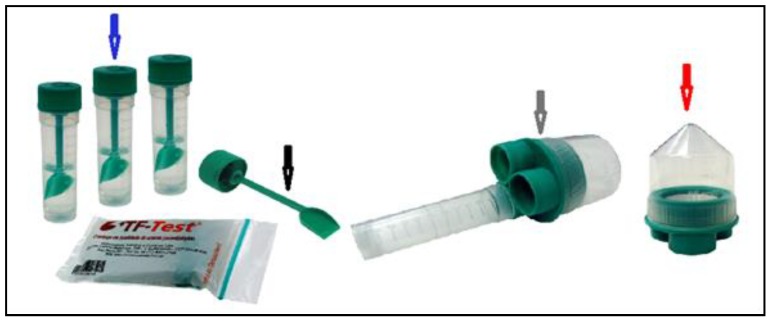
Image showing the components used in the Three Fecal Test (TF-Test) technique, the three collecting tubes containing preservative solution (blue arrow); collecting shovel (black arrow); filter set (gray arrow); and centrifuge tube (red arrow).

To investigate the parasite infections, fecal samples were collected in two steps, in triplicate, from each animal using appropriate TF-Test tubes, containing 5 mL of 7.5% formalin neutral solution each (Figure 2), which were then processed in the Faculty of Veterinary Medicine (FVM) laboratory of São Paulo State University (UNESP, acronym in Portuguese) in Araçatuba, SP.

In this study we used the technique known as TF-GII/Dog [3] and the Prototic Coproparasitological Test for Dogs (PC-TEST DOG) described below.

The samples collected were analyzed as follows: Step 1—Laboratory evaluation of the Parasitological Protocol (PC)-TEST DOG for the DAPI system and creation of an image database composed of parasites and fecal impurities; Step 2—The TF-GII/Dog technique [3] and the PC-TEST DOG protocol were compared against each other, and the same slides were tested in the DAPI system to evaluate the automated detection. The slides were evaluated by two parasitology specialists. 

### 2.2. Study Design

The first sampling step consisted of obtaining fecal samples from each one of the 104 dogs, on alternating days, using three TF-Test collection tubes (Figure 2, blue arrow). In the laboratory, the samples were processed following the protocols for the TF-Test technique (http://www.bio-brasil.com/produto/tf-test) and the PC-TEST DOG to determine the positive samples that were used to form the image database.

#### 2.2.1. TF-GII/Dog Protocol

Similar to the PC-TEST DOG protocol, triple fecal sampling was performed using the same collecting tubes. In the laboratory, the three tubes were homogenized by vigorous manual shaking. To the homogenized liquid material of each tube, 30 µL of nonionic surfactant and 3 mL of single ester ethyl acetate pro analysis were added, followed by new manual and vigorous homogenization, and each tube was then fitted to the filter.

The material was centrifuged at 500× *g* for 60 s. As previously described, the three collecting tubes were separated from the filter sets, which were discarded in an appropriate place for contaminant solids and liquids, according to Quality Control and Biosafety Standards. The supernatant material from the centrifuge tube was discarded in a suitable place, while 300 µL of water from reverse osmosis was added to the fecal sediment of this tube. The centrifuge tube was further homogenized, this time using a disposable plastic Pasteur pipette to form a fecal suspension. The suspension of this tube was transferred to a clean TF-Test tube without preservative liquid. 

To this suspension, 3 mL of zinc sulfate (ZnSO_4_), with 1.18 g/mL specific density, was added. This tube was further homogenized using a pipette, and it was supplemented with ZnSO_4_ until a meniscus formed on the top. A microscope slide was placed over the formed meniscus and left to stand for 15 min. After this, the slide was removed with a sudden inversion movement, stained, and taken for reading under conventional light microscopy.

#### 2.2.2. PC-TEST DOG Laboratory Protocol

The three collection tubes containing fecal samples from each animal were homogenized using a vortex mixer at 3000 rpm (g) for 30 s. To each tube, 25 µL of 10% sodium dodecyl sulfate (SDS) anionic surfactant and 3 mL of ethyl acetate (CH_3_COOC_2_H_5_) pro analysis were added. Afterward, the three tubes were homogenized again in identical conditions to obtain a uniform suspension of fecal particles.

The tubes were then individually assembled into the filter (Figure 2, gray arrow) to form the TF-Test Processor Assembly (PA), named PA/TF-Test, and centrifuged again at 500× *g* for 120 s. It is noteworthy that the PA/TF-Test is suitable for processing in a 100 mL universal centrifuge tube holder.

Subsequently, the filter was separated from the three centrifuge tubes and disposed of in an appropriate location for contaminant solids and liquids following the Quality Control and Biosafety Standards. The supernatant liquid from the centrifuge tube was decanted, allowing the fecal sediment to settle on the conical bottom of the tube (Figure 2, red arrow), to which 200 µL of water from reverse osmosis was added.

The tube was further homogenized, this time using a Pasteur disposable plastic pipette to form a fecal suspension. An aliquot of 300 µL of suspension was taken from this centrifuge tube and transferred to a clean, liquid-free TF-Test collection tube (Figure 2, blue arrow). To this tube, 3 mL of 7.5% neutral formalin solution and 3 mL of ethyl acetate were added, followed by another homogenization in the vortex mixer at 3000 rpm for 60 s. The supernatant liquid was decanted to form a fecal sediment on the bottom to which 50 µL of the 2.5% sodium hypochlorite chemical (NaClO) was added. Using the same vortex equipment, new homogenization was performed at 3000 rpm for 10 s. An aliquot of 20 µL of the fecal suspension was removed using an automatic micropipette and transferred to a microscope slide, to which 40 µL of water from reverse osmosis and 50 µL of 20% Lugol solution were added. Finally, a fecal smear was prepared on this slide for reading using conventional light microscopy.

#### 2.2.3. Study of Computational Techniques for Detecting Parasites (DAPI)

For the detection of parasites in the DAPI system, the image processing algorithm was divided into three main steps: image segmentation, object representation, and object recognition, as described below.

(a) In the image segmentation step, an image may contain several parasite structures such as eggs and cysts, and many impurities (fecal residues and adipose tissue) with size, shape, color, and texture similar to those of the parasites. An enhanced image is computed to increase the contrast and make parasites and some similar impurities brighter and the rest of the image darker. The enhancement explores differences between the color channels since red and green components tend to be higher than blue for the relevant objects. This image is thresholded, and the objects with area outside the range of parasites are eliminated, and the remaining ones are submitted to feature extraction.

(b) An object representation is based on color, shape, and texture. In this work, we used the pixel connectivity, object area, perimeter, symmetry, major and minor axes of the ellipse that best fit the object; and for classification they were used the difference between the ellipse and the object, energy, entropy, variance, and homogeneity of the co-occurrence matrix as measures to describe the objects.

(c) The object recognition step uses a Support Vector Machines (SVM) classifier trained to separate impurities and each of the 4 species of canine intestinal parasites. 

**Figure 3:**
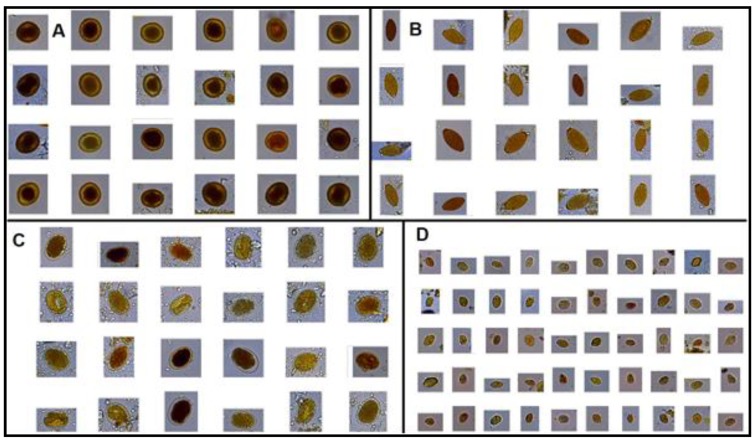
Images from the database showing four parasite genera (**A**) *Toxocara* spp. eggs; (**B**). *Trichuris* spp. eggs; (**C**) *Ancylostoma* spp. eggs; and, (**D**) *Giardia* spp. cysts.

**Figure 4:**
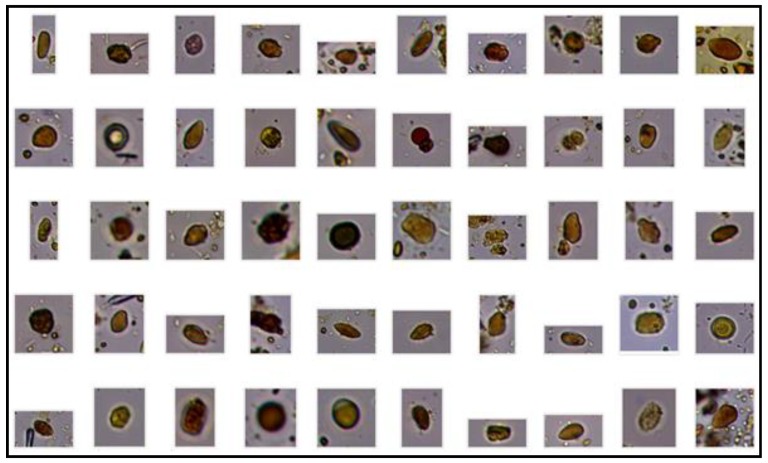
Images from the same database showing multiple fecal impurities.

To train the SVM classifier, we used a database consisting of 10,699 components, composed of 3132 parasite images (*Ancylostoma* spp.—155 eggs, *Trichuris* spp.—136 eggs, *Toxocara* spp.—277 eggs, and *Giardia* spp.—2564 cysts) and 7567 fecal impurities consisting primarily of cells, organic and plant structures of undigested digestive waste, according to Figure 3 and Figure 4.

### 2.3. Comparison between Parasitological Protocols 

In this work, the comparative evaluation stage was performed qualitatively using the reading of fecal smears prepared on microscope slides following the technical procedures of TF-GII/Dog and PC-TEST DOG. For comparison, the same slides prepared following the TF-GII/Dog and PC-TEST DOG protocols were evaluated by two parasitology specialists, and the same slides were evaluated by the two technicians blindly, using a 10× conventional light optical microscope for scanning and 40× to confirm the parasitic structure, as recommended in the literature [24]. 

#### 2.3.1. DAPI System

According to Section 2.2.2, automated reading of a microscope slide with fecal smear was executed using image processing algorithm for the DAPI system. The slide was prepared following the PC-TEST DOG protocol (Section 2.3.1) and placed on the motorized stage of the DAPI system (Figure 1), it was controlled by the computer commands for automated focus and scanning, and it was able to capture about 2000 image fields in approximately four minutes.

### 2.4. Parametric Statistical Analysis

In order to compare the proportion of positive parasite detection results between the mean obtained by the new PC-TEST DOG protocol and the TF-GII/Dog laboratory protocol, this study used parametric descriptive analyses using the McNemar test and Kappa coefficient of agreement (k), and the results were considered significant with *p* < 0.05. The k reference data were classified as poor (<0.00), slight (0–2.0), fair (0.21–0.40), moderate (0.41–0.60), substantial (0.61–0.80), and almost perfect (0.8–1.0) [30].

## 3. Results

Table 1 shows the comparison between TF-GII/Dog e PC-TEST DOG, and it shows that the PC-TEST DOG protocol (*p* < 0.0001) had 80.88% sensibility, with a substantial agreement (Kappa coefficient = 0.7636).

**Figure 5:**
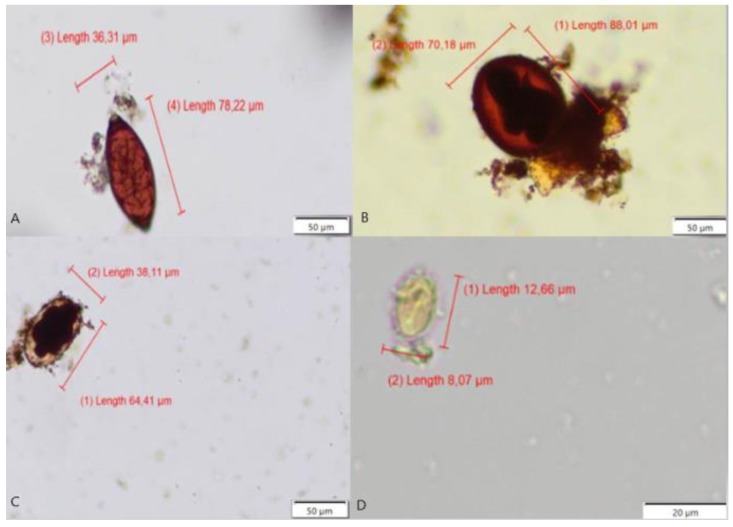
Images showing the TF-GII/Dog slides with parasites adhered to fecal impurities, (**A**) *Trichuris* spp. eggs; (**B**) *Toxocara* spp. eggs; (**C**) *Ancylostoma* spp. eggs; and, (**D**) *Giardia* spp. cyst.

The TF-GII/Dog technique showed a high number of impurities (Figure 5), as demonstrated by conventional techniques. 

**Figure 6:**
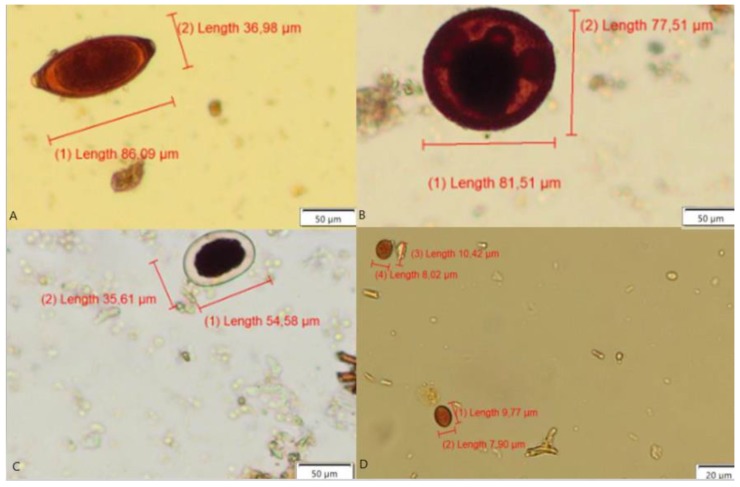
Images showing the Prototic Coproparasitological Test for Dogs (PC-TEST DOG) protocol processed slide with parasites free of fecal impurities, (**A**) *Trichuris* spp. eggs, (**B**) *Toxocara* spp. eggs, (**C**) *Ancylostoma* spp. eggs, and (**D**) *Giardia* spp. cysts.

On the other hand, Figure 6 shows that most of the smears prepared following the PC-TEST DOG protocol had no impurities attached to the parasites. 

In Table 1 a greater number of infected dogs showing the sensitivity 80.88% was found by the PC-TEST DOG technique, while the TF-GII/Dog technique showed 61.76%.

The statistical analysis shows that the PC-TEST DOG readings were 6.73% higher with 52.88% (55/104) average positive compared to 46.15% (48/104) of the DAPI system, indicating a significant difference (*p* < 0.0156) and an almost perfect Kappa agreement (k = 0.8660).

The data presented in this study demonstrated that the PC-TEST DOG, in partial agreement with DAPI, were able to identify efficiently four genera of intestinal parasites—*Giardia* spp. cysts and *Trichuris* spp., *Ancylostoma* spp. and *Toxocara* spp. eggs—present in the fecal matter of dogs (Table 2). 

## 4. Discussion

The new PC-TEST DOG protocol, allowing a better visualization of the parasitic structures, showed a substantial result (Kappa coefficient = 0.7636) (Table 1) for the diagnosis of intestinal parasites in dogs, especially regarding the egg and cysts identification of *Ancylostoma* spp. and *Giardia* spp., respectively.

The samples collected from the 104 dogs were satisfactory for the study using the new PC-TEST DOG protocol, and a parasite infection occurrence of 52.88% was detected for the animals housed in shelters located in Araçatuba, SP. 

The most frequently occurring parasites detected by the PC-TEST DOG technique in dogs were *Giardia* spp. and *Ancylostoma* spp. eggs. (Table 2), with a significant statistical difference (*p* < 0.0001). The findings of our study indicate that this expressive difference results from the fact that flotation principle techniques use high-saturation chemical reagents, such as 1.18 g/mL ZnSO_4_ for 10 to 15 min in TF-GII/Dog, which is able to decompose morphologically the parasites that have thin external lipoprotein membranes, like the two parasite genera mentioned above [22,24]. It is emphasized that the same reagent (ZnSO_4_) was used in this study without changing the egg morphology of the two parasites *Trichuris* spp. and *Toxocara* spp. that have thick membranes containing a bimolecular lipid stratum and a single protein (Figure 6A,B).

Figure 5 and Figure 6 show that the proposed PC-TEST DOG protocol allowed preparing microscope slides with fecal smears that showed the parasites mostly free of fecal residues, thus improving visualization. This new parasitological protocol freed the parasite structures from these residues (Figure 6), allowing a more efficient computational image segmentation technique while detecting clean parasite structures [27,28,29]. We concluded that the anionic surfactant SDS that is part of the PC-TEST DOG laboratory protocol acted on the parasite outer membranes, which have indicated to be negatively charged.

In the literature, several works concluded that the larger the image database, the more effective detection computational techniques become [27,28,29,30]. Currently, we have a small image database (10,699 components/data) to train this automated diagnostic system, which should be further extended as the study advances. 

In addition, the DAPI system significantly advanced the technique compared to several studies on automated diagnosis algorithms for human feces [31,32,33,34,35,36], since, to our understanding, these works largely evade a real examination process of feces by relying on perfect parasite images and are really good with respect to focus plane and color, and preferably with a residue-free parasite. The real examination has a mandatory protocol consisting of several steps, such as sampling, transport, and laboratory preparation of feces for later reading under conventional light microscopy [14,22,23,24]. 

Finally, this research provided significant diagnosis advances of examination in veterinary medicine, so that this knowledge can be further extended to the diagnosis of other species of canine parasites and to other species of small and large vertebrate animals as well.

The study and improvement of this new protocol, which has already demonstrated good performance in the diagnosis of dog intestinal parasites, would allow us to advance the development of automated diagnoses in veterinary medicine very similar to the DAPI system developed for parasitological diagnosis in humans [26], which requires a protocol that gets a cleaner microscope slide, free of impurities and debris, so that the computer system can identify parasitic structures more accurately.

## 5. Conclusions 

We concluded in this study that PC-TEST DOG in combination with DAPI is the better protocol for computational image segmentation while detecting clean parasites structures.

Diagnosis of canine intestinal parasites shows low to moderate diagnostic sensitivity. In this condition, we developed and evaluated an automated diagnostic system (DAPI) in a study involving four genera of canine parasites. Samples of 104 dogs were collected for this work using a new parasitological protocol, named PC-TEST DOG, which was evaluated and validated against a parasitological technique, TF-GII/Dog. A database composed of 10,699 components was created for researching the DAPI system, which was examined against the PC-TEST DOG results obtained by two parasitology specialists. Although this automated diagnostic system advanced the parasitological diagnosis, our findings indicate the need for further research to create a more robust database with better images showing parasites free of impurities that should be used in the image analysis techniques based on segmentation and pattern classification.

## Figures and Tables

**Table 1:** Parasites detected by TF-GII/Dog, PC-TEST DOG techniques and the gold standard, showing the sensitivity of each technique.

Results	TF-GII/Dog	PC-TEST DOG	Total Cases (GS)
TP	42	55	68
TN	42	42	42
FN	26	13	0
FP	0	0	0
Sensibility	61.76%	80.88%	0
Specificity	100.00%	100.00%	
Accuracy	76.36%	88.18%	
*kappa*	0.55	0.76	
Agreement	Moderate	Substantial	

Legend: TP (True Positive); TN (True Negative); FN (False Negative); FP (False Positive) and GS (Gold Standard).

**Table 2:** Number of parasites detected by TF-GII/Dog and PC-TEST DOG techniques.

Parasite	TF-GII/DOG	PC-TEST DOG	DAPI System
* Giardia spp.	16 (15.38%)	28 (26.92%)	28 (26.92%)
* Ancylostoma spp.	11 (10.57%)	17 (16.34%)	12 (11.54%)
*Trichuris* spp.	2 (1.92%)	2 (1.92%)	2 (1.92%)
*Toxocara* spp.	13 (12.50%)	8 (7.69%)	6 (5.77%)
Total Positive	42 (40.38%)	55 (52.88%)	48 (46.15%)
Total Negative	62 (59.61%)	49 (47.12%)	56 (53.85%)
Total dogs	104 (100%)	104 (100%)	104 (100%)

Legend: * (*p* < 0.0001) through the PC-TEST DOG technique.
